# *Streptomyces* spp. Strains as Potential Biological Control Agents against Verticillium Wilt of Olive

**DOI:** 10.3390/jof10020138

**Published:** 2024-02-08

**Authors:** Miriam Díaz-Díaz, Begoña I. Antón-Domínguez, María Carmen Raya, Alexander Bernal-Cabrera, Ricardo Medina-Marrero, Antonio Trapero, Carlos Agustí-Brisach

**Affiliations:** 1 Centro de Bioactivos Químicos (CBQ), Universidad Central “Marta Abreu” de Las Villas (UCLV), Carretera Camajuaní km 5 1/2, Santa Clara 54830, Villa Clara, Cuba; miriamdd@uclv.cu (M.D.-D.); rpmedina@uclv.edu.cu (R.M.-M.); 2 Departamento de Agronomía, (Unit of Excellence ‘María de Maeztu’ 2020–2024), Universidad de Córdoba, Campus de Rabanales, Edif. C4, 14071 Córdoba, Spain; z72andob@uco.es (B.I.A.-D.); ag2raorm@uco.es (M.C.R.); ag1trcaa@uco.es (A.T.); 3 Centro de Investigaciones Agropecuarias (CIAP), Facultad de Ciencias Agropecuarias, Universidad Central “Marta Abreu” de Las Villas (UCLV), Carretera Camajuaní km 5 1/2, Santa Clara 54830, Villa Clara, Cuba; alexanderbc@uclv.edu.cu; 4 Departamento de Agronomía, Facultad de Ciencias Agropecuarias, Universidad Central “Marta Abreu” de Las Villas (UCLV), Carretera Camajuaní km 5 1/2, Santa Clara 54830, Villa Clara, Cuba

**Keywords:** actinomycetes, biocontrol, *Olea europaea*, soilborne pathogen, *Verticillium dahliae*

## Abstract

Verticillium wilt of olive (VWO) caused by *Verticillium dahliae* is considered a major olive (*Olea europaea*) disease in Mediterranean-type climate regions. The lack of effective chemical products against VWO makes it necessary to search for alternatives such as biological control. The main goal of this study was to evaluate the effect of six *Streptomyces* spp. strains as biological control agents (BCAs) against VWO. All of them were molecularly characterized by sequencing 16S or 23S rRNA genes and via phylogenetic analysis. Their effect was evaluated in vitro on the mycelial growth of *V. dahliae* (isolates V004 and V323) and on microsclerotia (MS) viability using naturally infested soils. Bioassays in olive plants inoculated with *V. dahliae* were also conducted to evaluate their effect against disease progress. In all the experiments, the reference BCAs *Fusarium oxysporum* FO12 and *Aureobasidium pullulans* AP08 were included for comparative purposes. The six strains were identified as *Streptomyces* spp., and they were considered as potential new species. All the BCAs, including *Streptomyces* strains, showed a significant effect on mycelial growth inhibition for both *V. dahliae* isolates compared to the positive control, with FO12 being the most effective, followed by AP08, while the *Streptomyces* spp. strains showed an intermediate effect. All the BCAs tested also showed a significant effect on the inhibition of germination of *V. dahliae* MS compared to the untreated control, with FO12 being the most effective treatment. Irrigation treatments with *Streptomyces* strain CBQ-EBa-21 or FO12 were significantly more effective in reducing disease severity and disease progress in olive plants inoculated with *V. dahliae* compared to the remaining treatments. This study represents the first approach to elucidating the potential effect of *Streptomyces* strains against VWO.

## 1. Introduction

The hemibiotrophic soil-borne fungus *Verticillium dahliae* Kleb. is considered to be among the main pathogens that affect cultivated olive (*Olea europaea* subsp. *Europaea* L.). It is the causal agent of Verticillium wilt of olive (VWO) in the main olive-growing regions of the Mediterranean basin, including Spain. The disease has important effects on fruit yield reduction as well as on plant viability [1]. The Andalusia region (southern Spain) represents 62.45% of the Spanish olive-growing area (2,635,276 ha) [2]. In this region, two pathotypes of *V. dahliae* have been identified as causing VWO: (i) the non-defoliating pathotype (ND) with low levels of severity without defoliation; and (ii) the defoliating pathotype (D) that causes the most severe symptomatology in the affected olives, including defoliation, xylem discoloration, and tree death [3]. The pathogen produces resting structures called microsclerotia (MS), which allow the pathogen to survive in the plant debris and soil for a long time. These structures are the primary inoculum sources that infect olives through the roots because of biochemical stimuli induced by the root exudates [1,4].

The difficulty in controlling VWO due to the interaction of several factors, such as the ability of the pathogen to produce MS in plant debris and in the soil, the lack of effective chemical treatments, the inadequate agronomic practices, or the use of homogeneous olive genotype populations that induce the pathogen to overcome the genetic resistance, among other factors, is well known [1,3,5]. Considering this scenario, the need to establish an integrated management strategy to control the disease based on eco-friendly control methods, including cultural practices, genetic resistance, and biocontrol, is an urgent demand in our society.

Regarding the biological control and bioprotection of VWO, important advances have been conducted during the last decade. A broad diversity of natural compounds such as plant extracts [6,7]; organic amendments [8,9]; biostimulants and biofertilizers [5]; and beneficial microorganisms, including bacteria, e.g., *Bacillales* members [10], *Bacillus amyloliquefaciens* [5,11], *Bacillus velezensis* [12,13], *Paenibacillus alvei* [14], *Pseudomonas* spp. [15], *Pseudomonas fluorescens* [16,17], and *Pseudomonas simiae* [18], and fungi, e.g., *Aureobasidium pullulans* AP08 [5,11,19], the non-pathogenic strain *Fusarium oxysporum* FO12 [20], and *Trichoderma* spp. [21,22,23,24,25] have been evaluated against VWO under controlled conditions, with some of them emerging as potential biological control agents (BCAs). However, only a few of them have already been evaluated in the field with successful results in reducing disease severity (DS), with the non-pathogenic *F. oxysporum* strain FO12, a *Thymus* extract, and a grape marc compost being the most effective [26].

The difficulty of obtaining effective BCAs against VWO makes the search for new biological alternatives necessary. In this way, *Streptomyces* species (Actinomycetes: Streptomycetales) are considered soil-borne bacteria that colonize the rhizosphere, promoting plant growth [27,28], and they can produce antibiotic substances that inhibit the growth of plant pathogenic fungi [29]. Several *Streptomyces* species have been described as plant growth promoters alongside their effectiveness against plant diseases [30,31,32].

The effect of *Streptomyces* species against *V. dahliae* viability has already been reported. The strains *S. cyaneofuscatus* ZY153 and AA-2, *S. flavotricini* Z-13, *S. kanamyceticus* B-49, *S. plicatus* 101, *S. rochei* X-4, and *Streptomyces* sp. StB-3, StB-6, StB-11, and StB-12 have inhibited *V. dahliae* mycelial growth through the in vitro activity of cell-free culture filtrates [33,34,35]. Xue et al. [35] observed that *Streptomyces* hyphae coiled around the hyphae of *V. dahliae* and demonstrated that the strains *S. cyaneofuscatus* ZY-153, *S. kanamyceticu* B-49, *S. rochei* X-4, and *S. flavotricini* Z-13 significantly reduced disease severity in cotton plants under both greenhouse and field conditions compared to the untreated controls. More recently, Calvo-Peña et al. [36] identified albocycline, a macrolide polyketide with antibacterial and antifungal activity, as a bioactive compound produced by *Streptomyces* sp. OR6 that was able to suppress the germination of *V. dahliae* conidia. In addition, the effect of *Streptomyces* strains against other soilborne pathogens has been tested. Errakhi et al. [37] evaluated several strains of *Streptomyces* spp. against *Botrytis cinerea*, *F. oxysporum*, *Sclerotium rolfsii*, and *V. dahliae* in vitro via double confrontation or culture filtration and in vivo on *Beta vulgaris*. In this study, the *Streptomyces* strains J-2 and B-11 inhibited 100% of sclerotial germination and 80% of fungal hyphal growth compared to the positive controls. Yang et al. [38] tested the fermentation broth of *S. corchorusii* strain AUH-1, which significantly inhibited the mycelial growth of *Fusarium oxysporum* f. sp. *niveum*, *Phytophthora capsica*, *Phytophthora parasitica* var. *nicotianae*, and *Rhizoctonia solani* compared to the control. Finally, Carlucci et al. [39] showed that the strain *S. albidoflavus* CARA17 inhibited the mycelial growth of *F. oxysporum*, *Plectosphaerella ramiseptata*, *Sclerotinia sclerotiorum*, *Sclerotium rolfsii*, and *V. dahliae*, with the highest inhibition (100%) being observed against *S. sclerotiorum*.

Likewise, there are no studies on the effectiveness of *Streptomyces* spp. against VWO. Therefore, the main goal of this study was to evaluate the effectiveness of *Streptomyces* spp. strains against VWO. To do this, the effects of six *Streptomyces* spp. strains on mycelial growth and on MS germination of *V. dahliae* were evaluated via dual cultures and sensitivity tests in naturally infested soils. Their effectiveness with respect to disease development was also evaluated by means of bioassays in olive plants inoculated with *V. dahliae* under semi-controlled conditions. In all the experiments, the non-pathogenic strains *F. oxysporum* FO12 [20] and *A. pullulans* AP08 [5] were included for comparative purposes. In parallel, the *Streptomyces* strains were characterized molecularly by sequencing 16S and 23S rRNA genes and via phylogenetic analysis. This study introduces a further step in the biocontrol of VWO by exploring the effectiveness of *Streptomyces* spp. strains for the first time against this disease. These beneficial microorganisms could emerge as potential BCAs to prevent *V. dahliae* infections in olive trees.

## 2. Materials and Methods

### 2.1. Fungal Isolates and Culture Conditions

The *V. dahliae* isolates V323 (D pathotype) [40] and V004 (ND pathotype) [41] were used for this study. These isolates are maintained in the fungal collection of the Department of Agronomy at the University of Cordoba (UCO, Córdoba, Spain).

Prior to carrying out the studies described below, fresh colonies of the two *V. dahliae* isolates were obtained from the collection and grown on potato dextrose agar (PDA; Difco^®^ Laboratories, Sparks, MD, USA) acidified with lactic acid (APDA, 0.01% (*v*/*v*; pH = 4.0–4.5) to avoid bacterial contaminations and incubated at 24 ± 2 °C in the dark for 10 days. Fresh colonies were transferred to PDA, incubated as described before, and then used as the inoculum source.

### 2.2. Biological Control Agents and Inoculum Preparation

Six *Streptomyces* strains (CBQ-B-8, CBQ-CB-14, CBQ-CD-24, CBQ-EA-2, CBQ-EA-12, and CBQ-EBa-21) belonging to the collection of the Microbiology Laboratory of the “Centro de Bioactivos Químicos” (CBQ) of the Central University “Marta Abreu” of Las Villas (Cuba) were tested in this study. All of them were previously characterized according to their cellulolytic, chitinolytic, and proteolytic extracellular enzymatic activity, as well as by their morphological and biochemical characters [42]. They are preserved at −80 °C in 20% glycerol. Prior to conducting the experiments, the strains were grown on casein-starch agar (CSA; pH = 7.0) at 30 °C for 7 days in the dark. For further experiments, inoculum was prepared in liquid medium as described by Díaz-Díaz et al. [42].

Additionally, the non-pathogenic strain *F. oxysporum* FO12 [20] and *A. pullulans* AP08 [5] were included for comparative purposes since they were previously characterized as promising BCAs against VWO. Both isolates belong to the fungal collection of the Department of Agronomy (UCO, Spain). Growth conditions and inoculum preparation for *F. oxysporum* FO12 and *A. pullulans* AP08 were conducted as described by Mulero-Aparicio et al. [20] and López-Moral et al. [5], respectively.

### 2.3. Molecular Characterization of Streptomyces Strains

#### 2.3.1. DNA Extraction, PCR Analysis, and Sequencing

The six *Streptomyces* strains were grown on CSA as described above. DNA was extracted from the resulting pellet using the NucleoSpin^®^ Microbial DNA (MACHEREY-NAGEL GmbH and Co. KG, Düren, Germany) following the manufacturer’s instructions. To determine the concentration and purity of the extracted DNA, we used a MaestroNano^®^ spectrophotometer (MaestroGen, Hsinchu, Taiwan). 

The 16S rRNA gene was amplified by PCR using the universal primers pair 27F and 1492R [43,44,45]; the 23S rRNA gene was amplified using the universal primers pair 1643F and 1705R (with numbering based on *Streptomyces ambofaciens* position) [46,47]. For both genes, each PCR reaction mixture contained each primer at 0.4 µM, 1× MyTaq Reaction Buffer, 0.5 µL MyTaq DNA Polymerase (MyTaq^TM^ DNA Polymerase, Bioline, London, UK), and 20 ng of genomic DNA in a final volume of 25 µL. The PCRs cycling program included an initial denaturation at 94 °C for 1 min, followed by 35 cycles at 94 °C for 30 s, 50 °C (primers pair 27F/1492R) or 45 °C (primers pair 1643F/1705R) for 30 s, and 72 °C for 30 s, as well as a final extension step at 72 °C for 7 min. PCRs were stopped at 4 °C. A negative control, using ultrapure water instead of DNA, was included in all PCRs. These PCRs were carried out in a MyCycler™ Thermal Cycler (BIO-RAD, Hercules, CA, USA). PCR products were checked by 1.7% agarose gel electrophoresis stained with RedSafe™ dye (iNtRON Biotechnology, Seoul, Republic of Korea) and visualized under UV; the amplicons were then purified using the PureLink™ PCR purification kit (Invitrogen Life Technologies, Carlsbad, CA, USA). Sequencing was carried out in both directions by the Central Service Support Research (SCAI) of the UCO (Spain), and the resulting sequences were compared with those by BLAST^®^ in the GenBank database (https://blast.ncbi.nlm.nih.gov/Blast.cgi) to identify closely related sequences. 

#### 2.3.2. Phylogenetic Analysis

Consensus sequences were obtained from DNA sequences generated with forward and reverse primers using SeqMan software (DNASTART Lasergen SeqMan version 7.0.0, Madison, WI, USA). They were compiled into a single FASTA file format and deposited in GenBank. The GenBank accession numbers are shown in Table 1.

Two separate phylogenetic analyses were conducted to identify the *Streptomyces* strains by means of 16S or 23S rRNA gene sequences. A total of 31 or 32 taxa were included for the phylogenetic analysis with 16S or 23S rRNA genes, respectively, from which 6 taxa were our *Streptomyces* strains and the remaining taxa were GenBank reference sequences selected for their high similarity with our query sequences using BLAST as described above (Table 1). *Streptomyces caviscabies* strain 8 and *S. scabies* strain 87.79 were used as the outgroup for the 16S and 23S phylogenies, respectively (Table 1). In both cases, GenBank sequences were added to the sequences obtained and aligned via Clustal W. Afterwards, a maximum parsimony (MP) analysis was performed by the tree bisection and regrafting algorithm with search level one in which the initial trees were obtained via the random addition of sequences (10 replicates). Adding sequences, alignments, and MP analyses were performed using MEGA version 11 software [48]. Gaps and missing data were treated as complete deletions. The robustness of the topology was evaluated via 2000 bootstrap replications [49]. Measures for the MP analyses (tree length (TL), consistency index (CI), retention index (RI), homoplasy index (HI), and rescaled consistency index (RC)) were calculated for the resulting tree. 

### 2.4. Effect of *Streptomyces* spp. on *Verticillium dahliae* Viability

#### 2.4.1. Effect on Mycelial Growth

The antagonism of the six *Streptomyces* strains was evaluated against two *V. dahliae* isolates (V323 and V004) by means of a dual-culture assay. The antagonist strains FO12 and AP08 were included for comparative purposes. A mycelial agar plug (7.5 mm diameter) was removed from the edge of a 7-day-old actively growing colony of the pathogen and seeded in the center of a Petri dish (9.0 cm diameter) filled with PDA. Subsequently, two mycelial plugs (7.5 mm diameter) obtained from the margin of 7-day-old colonies of each *Streptomyces* strain, FO12 or AP08, were then placed on the same PDA plate but, on both sides of the pathogen, not further than 3 cm from the *V. dahliae* plug. PDA Petri dishes inoculated with only *V. dahliae* were included as control. For each *V. dahliae* isolate, there were three replicated Petri dishes per BCA or control arranged in a completely randomized design ((8 biological treatments + 1 control) × 2 *V. dahliae* isolates × 3 replicated Petri dishes = 54 Petri dishes in total). The experiment was conducted twice.

The Petri dishes were incubated at 28 °C for 14 days. The largest and smallest diameter of *V. dahliae* colonies were measured at 14 days after confrontation. The mean data were converted to obtain the mycelial growth rate (MGR, mm day^−1^), and the mycelial growth inhibition percentage (mycelial growth inhibition, MGI (%)) was calculated [5].

#### 2.4.2. Effect on Microsclerotia Viability

A natural infested soil was collected from an olive orchard affected by VWO located in Villanueva de la Reina (Jaen province, Andalusia region, southern Spain; Geographic UTM coordinates X: 38.0140220, Y: −3.9100390), previously grown with cotton (*Gossypium hirsutum*) for many years. Cotton is one of the most important herbaceous crops in Andalusia (southern Spain), where we find the 99% of the national cultivated surface of this crop [2]. The soil was manually sieved using a 0.8 mm diameter sieve to remove organic debris and large particles and thoroughly mixed in order to ensure uniform distribution of MS [50]. 

Sterile PVC pots (100 mL vol.) were used, and five holes of 2 mm in diameter were made in their bases to facilitate percolation. In each pot, 60 g of soil was added and immediately irrigated with 30 mL of conidial suspension of each treatment. For this experiment, the six *Streptomyces* strains and a mix of the *Streptomyces* strains CBQ-EA-2 and CBQ-B-8 were tested. FO12 and AP08 were included for comparative purposes. Conidial suspensions of *Streptomyces* strains were adjusted at 10^8^ spores mL^−1^. In addition, a suspension mixing the *Streptomyces* strains CBQ-EA-2 and CBQ-B-8 was also prepared and adjusted to a final concentration of 10^8^ spores mL^−1^ of each strain. Conidial suspensions of FO12 or AP08 were adjusted at 10^6^ conidia mL^−1^ based on previous studies [5,20]. All conidial suspensions were adjusted according to hemocytometer counts. In addition, pots with 60 g of soil irrigated with sterile distilled water were included as control. After treatment percolation, the pots were hermetically closed and incubated for 48 h at room temperature. Then, treated soils were placed in individual aluminum trays and air-dried at room temperature for 10–14 days [40]. A completely randomized design with three replicated pots per treatment or control was used ((9 biological treatments + 1 control) × 3 replicated PVC pots = 30 PVC pots in total). The experiment was conducted twice.

The inoculum density of *V. dahliae* was expressed as MS per g of soil for each soil sample. It was assessed via the wet sieving method [51] using 10 Petri dishes with modified sodium polypectate agar (MSPA) [52] according to the protocol described by Varo et al. [19]. Subsequently, the reduction in viable inoculum was estimated with respect to the control and expressed as MS inhibition (MSI, %) [5].

### 2.5. Effect of *Streptomyces* spp. on Disease Development in Olive Plants

#### 2.5.1. Plant Material and Growth Conditions

Nine-month-old healthy and untreated olive potted plants of cv. Picual (highly susceptible to *V. dahliae*) [53] were obtained from a commercial nursery. Plants were grown in 0.5 L PVC pots containing commercial peat moss, and the plant material and substrate were certified as free of *V. dahliae*. Prior to conducting the experiments, plants were preconditioned in environmentally controlled chambers at 22 ± 2 °C with a 14:10 h (light:dark) photoperiod of white fluorescent light (10,000 lux) and 60% relative humidity (RH) for 1 month before starting the experiments, and they were watered three times a week [5].

#### 2.5.2. Biological Control Agents

The six *Streptomyces* strains and the mix of *Streptomyces* strains CBQ-EA-2 and CBQ-B-8 were evaluated. The fungal strains FO12 and AP08 were included for comparative purposes. The biological treatments were applied by irrigating the olive plants with 250 mL of conidial suspensions at different times (see below). Conidial suspensions were prepared as described above and adjusted at 10^8^ spores mL^−1^ for *Streptomyces* and 10^6^ conidia mL^−1^ for FO12 and AP08 using a hematocytometer.

#### 2.5.3. *Verticillium dahliae* Isolate and Inoculum Preparation

The *V. dahliae* isolate V323 was used for this experiment. The inoculum was prepared in 2 L Erlenmeyer flasks filled with 1 kg of a homogeneous mixture consisting of sand, maize flour, and distilled water (9:1:2, *w:w:v*). Each flask was double-sterilized first at 120 °C for 50 min (1st day), then 24 h later at 120 °C for 20 min (2nd day), and manually shaken after each sterilization process. Then, 50 mycelial plugs (7.5 mm in diameter) of *V. dahliae* isolate V323, growing on PDA as described above, were introduced into each flask. The flasks were incubated at 24 °C in the dark for 4 weeks, and they were manually shaken just after inoculation and once a week during the incubation period [5]. Just before conducting the inoculation, the inoculum density of the colonized mixture was assessed via the serial dilution method on PDA and adjusted mixing the inoculum with sterile peat moss to 10^7^ colony-forming units (CFU)/g in order to confirm that there was a uniform distribution of inoculum in this experiment [9].

#### 2.5.4. Biological Treatments and *V. dahliae* Inoculation

Biological treatments were applied by irrigating the olive plants with 250 mL of the adjusted conidial suspensions at three different moments, i.e., at 7 and 2 days before inoculation with *V. dahliae* and at 10 days after inoculation. At the inoculation time, olive plants were uprooted from the original pot, the original peat moss was carefully removed, and bare roots were cleaned under running tap water. Then, plants were transplanted to 0.8 L PVC pots previously disinfested in a 10% sodium hypochlorite solution and filled with a 20% (*w*/*w*) mixture of the previously described inoculum and sterile peat moss, obtaining a theoretical inoculum density of the final substrate of 10^7^ CFU g^−1^ [20]. Additionally, untreated and inoculated and untreated and non-inoculated (20% (*w/w*) mixture of sterile corn meal-sand and sterile peat moss) plants were included as positive and negative controls, respectively. All plants were incubated in a growth chamber at 19 °C in the dark and 100% RH for 3 days after inoculation. Subsequently, light and RH parameters were progressively modified over 1 week until reaching 23 °C, a 12 h photoperiod of fluorescent light (10,000 lux), and 70% RH, which were maintained until the end of the experiment. The plants were watered three times a week.

A randomized complete block design (three blocks) was used with nine biological treatments and two controls (positive and negative) as independent variables and five replicated olive plants per treatment and block (11 × 3 × 5 = 165 olive plants). The experiment was conducted twice.

#### 2.5.5. Disease Severity Assessment

DS was assessed according to the percentage of affected plant tissues (leaves and shoots) showing symptoms of chlorosis, necrosis, and/or defoliation. The plants were evaluated weekly for 16 weeks by means of a 0–16 severity rating scale that is subdivided into four categories (0–25, 26–50, 51–75, and 76–100% of affected tissue), with four values per category (0.25, 0.5, 0.75, and 1.0) [8]. DS data were used to calculate the relative area under the disease progress curve (RAUDPC) by means of the trapezoidal integration method [54]. The final DS was recovered for further analysis, and the disease incidence (DI; % of affected plants) and mortality (% of dead plants) were estimated at the end of the experiment as the percentage of symptomatic or dead plants, respectively. 

Three symptomatic plants per treatment or control were randomly selected at the end of the experiment to confirm the infection of the pathogen by fungal isolation. Basal stems of the plants were washed under running tap water for 2 h. Subsequently, small fragments of the affected tissue were cut, surface-sterilized by dipping them in a 10% solution of commercial bleach (Cl at 50 g L^−1^) for 1 min, air-dried on sterilized filter paper for 10 min, and plated onto APDA. Petri dishes were incubated as described above, and the frequency of isolation of *V. dahliae* was estimated as the percentage of pieces in which the pathogen was isolated compared to the total pieces used for isolation (%).

### 2.6. Data Analysis

Data from the two repetitions of each experiment were combined after checking for homogeneity of the experimental error variances via the *F* test (*p* ≥ 0.05). In all cases, data were tested for normality, homogeneity of variances, and distribution pattern of the residuals. For dual culture assay, a factorial ANOVA was conducted with MGI as the dependent variable, and BCAs, *V. dahliae* isolates, and their interaction as independent variables. Because significant differences were observed for BCAs (*p* ≤ 0.0001) and *V. dahliae* isolates (*p* = 0.0002) but not for their interaction (*p* = 0.0919), a global one-way ANOVA was conducted for the two *V. dahliae* isolates with MGI as the dependent variable and BCAs as the independent variable. For MS viability, a one-way ANOVA was conducted with MSI as the dependent variable and BCAs as independent variables. For the in planta experiment, one-way ANOVA was conducted with RAUDPC or final DS as dependent variables and BCAs as the independent variable. In all cases, treatments that did not show symptoms in any of the plants were excluded; and treatment means were compared according to Fisher’s protected LSD test at *p* = 0.05 [55]. Data of DI (% of affected plants) and mortality (% of dead plants) were analyzed via multiple comparisons for proportions tests at *p* = 0.05 [56]. Data from this study were analyzed using Statistix 10.0 software [57].

## 3. Results

### 3.1. Molecular Characterization of Streptomyces Strains

**Figure 1:**
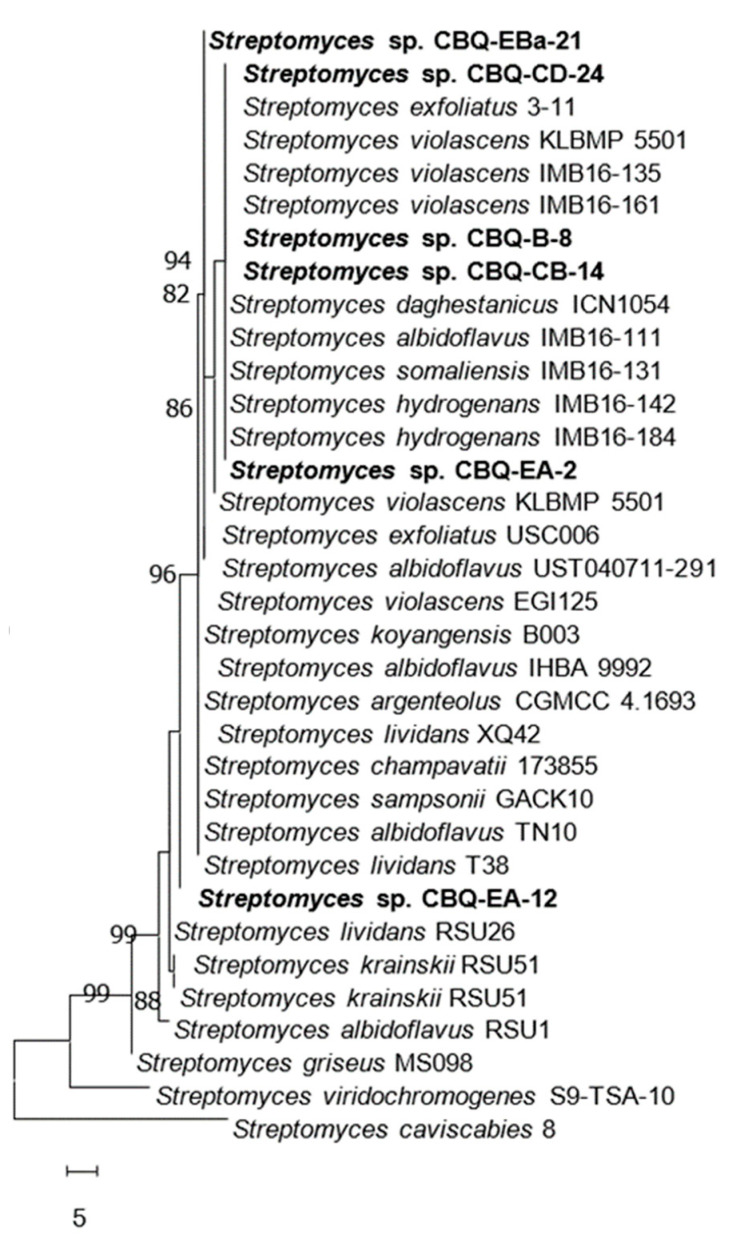
Evolutionary relationships of *Streptomyces* spp. based on the 16S rRNA gene sequences. The evolutionary history was inferred via the maximum parsimony test. One of the five most parsimonious trees is shown (TL = 88; CI = 0.926; RI = 0.980; HI = 0.074; RC = 0.907). Bootstrap support values (MP > 70%) are shown at the nodes (2000 replications). *Streptomyces caviscabies* strain 8 was used as the outgroup. Evolutionary analyses were conducted in MEGA11 [48]. Isolates from the current study are indicated in bold.

**Figure 2:**
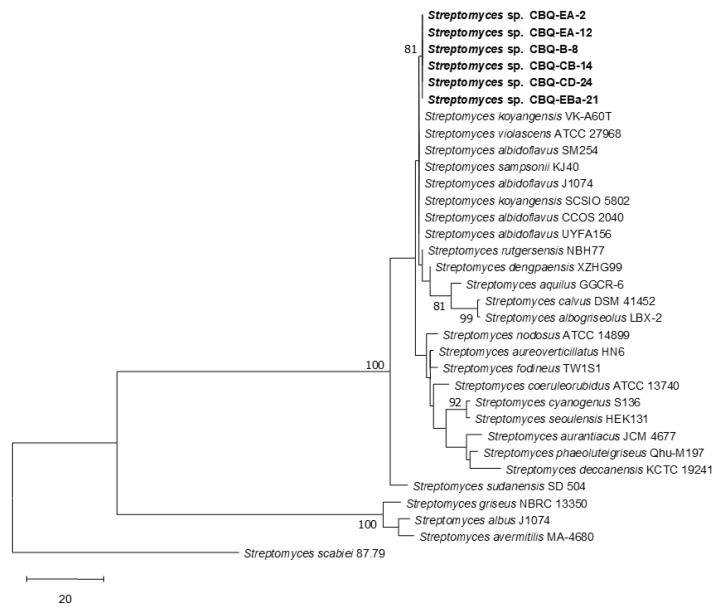
Evolutionary relationships of *Streptomyces* spp. based on the 23S rRNA gene sequences. The evolutionary history was inferred by the maximum parsimony test. One of the five most parsimonious trees is shown (TL = 320; CI = 0.844; RI = 0.890; HI = 0.156; RC = 0.751). Bootstrap support values (MP >70%) are shown at the nodes (2000 replications). *Streptomyces scabiei* strain 87.79 was used as the outgroup. Evolutionary analyses were conducted in MEGA 11 [48]. Isolates from the current study are indicated in bold.

Comparative analysis of the 16S and 23S rRNA gene sequences of our *Streptomyces* strains confirmed that all they belong to the genus *Streptomyces.* The similarity of our strains ranged from 97.00 to 99.78% when they were compared with the reference sequences available in the NCBI database. For both 16S and 23S alignments, our *Streptomyces* strains did not cluster in well-supported clades with reference sequences of specific *Streptomyces* species. In the 16S alignment, our six *Streptomyces* strains formed part of a monophyletic group since the strains CBQ-B-8, CBQ-CB-14, CBQ-CD-24, CBQ-EA-2, CBQ-EA-12, and CBQ-EBa-21 were grouped in a subclade (Bootstrap support, BS = 82%) with reference sequences of *S. albidoflavus*, *S. daghestanicus*, *S. exfoliatus*, *S. hydrogenans*, *S. somaliensis*, *S. violascens*, and *Streptomyces* sp.; the strain CBQ-EA-12 was located individually in a clade, which was the sister clade of the clade formed by the previous subclade, with reference sequences of *S. albidoflavus*, *S. argenteolus*, *S. champavatii*, *S. koyangensis*, *S. lividans*, *S. sampsonii*, and *S. violascens* (Figure 1). Regarding the 23S alignment, our six *Streptomyces* strains clustered in a heterogeneous clade with reference sequences of *S. albidoflavus*, *S. albogriseolus*, *S. aquilus*, *S. calvus*, *S. dengpaensis*, *S. koyagensis*, *S. rutgersensis*, *S. sampsoni*, and *S. violascens* (Figure 2). Because none of the reference sequences belonging to the *Streptomyces* genus matches with sequences of our strains with high a similarity percentage, these were considered unidentified species belonging to the *Streptomyces* genus (*Streptomyces* sp.)

### 3.2. Effect of *Streptomyces* spp. on *Verticillium dahliae* Viability

#### 3.2.1. Effect on Mycelial Growth

**Figure 3:**
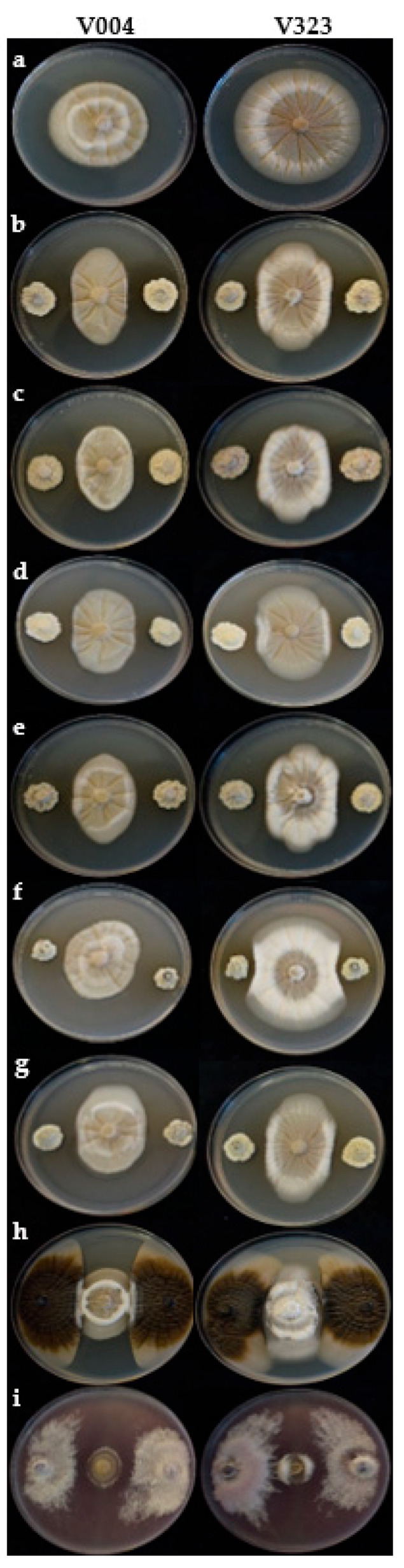
Antagonistic effect of BCAs against *Verticillium dahliae* in dual cultures growing on PDA at 28 °C for 14 days in the dark. The control or treatments are arranged in a row as follows: (**a**) control of *V. dahliae* isolates V004 (**left**) and V323 (**right**); (**b**–**g**) *Streptomyces* spp. strains; (**b**) CBQ-B-8; (**c**) CBQ-CB-14; (**d**) CBQ-CD-24; (**e**) CBQ -EA-2; (**f**) CBQ -EA-12; (**g**) CBQ -EBa-21; (**h**) *Aureobasidium pullulans* AP08; and (**i**) *Fusarium oxysporum* FO12.

The six *Streptomyces* sp. strains and the fungal strains FO12 and AP08 showed a significant effect on MGI for both *V. dahliae* isolates V004 and V323 (*p* ≤ 0.001 in all cases) compared to the positive control. For all BCA treatments, *V. dahliae* isolates showed significant differences in mycelial growth development, with V323 growing more than V004. The reference BCA FO12 (76.4 ± 1.87%) was the most effective in terms of MGI, followed by AP08 (52.1 ± 1.83%); while the *Streptomyces* sp. strains showed a moderate effect. Among the *Streptomyces* strains, CBQ-EA-2, CBQ-B-8, and CBQ-CB-14 were the most effective in terms of MGI, while the CBQ-EA-12 strain was the least effective, with MGI ranging from 44.4 ± 1.67 to 33.6 ± 2.91% for *Streptomyces* sp. CBQ-EA-2 and CBQ-EA-12, respectively (Figure 3; Table 2). 

#### 3.2.2. Effect on Microsclerotia Viability

**Figure 4:**
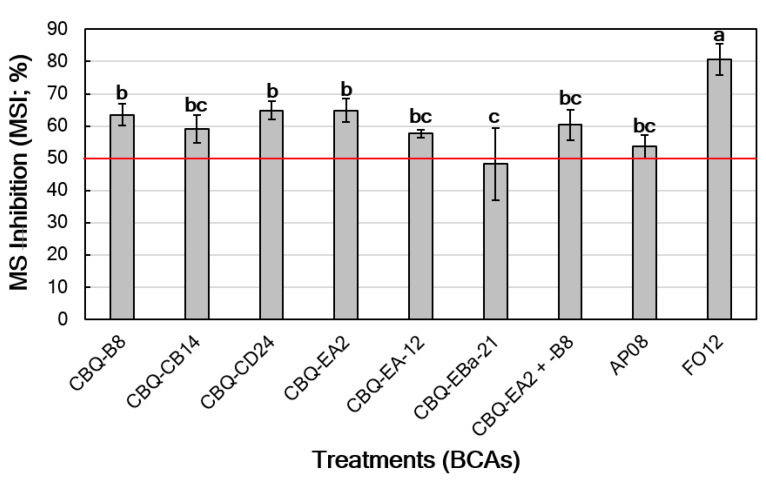
Effect of BCAs on the viability of *Verticillium dahliae* microsclerotia (MS inhibition, MSI (%)) in naturally infested soil. Columns represent the average of six replicated PVC pots, and columns with a common letter do not differ significantly according to Fisher’s protected LSD test at *p* = 0.05 [55]. Vertical bars represent the standard error of the means. Red line indicates the 50% MSI threshold.

All the BCAs tested showed a significant effect on the inhibition of germination of *V. dahliae* MS (*p* = 0.0218) compared to the untreated control. All the *Streptomyces* treatments showed MSI values above 50% except for CBQ-EBa-21 (MSI = 48.2 ± 11.3%). MSI values for *Streptomyces* treatments ranged from 57.7 ± 1.2 to 64.9 ± 2.8 or 64.9 ± 3.6% for *Streptomyces* sp. strains CBQ-EA-12 and CBQ-CD-24 or CBQ-EA2, respectively. All the *Streptomyces* treatments showed a similar effect as that observed for AP08 (MSI = 53.6 ± 3.6%); while FO12, significantly showed the highest effectiveness in terms of MSI (MSI = 80.6 ± 4.8%) compared to the remaining treatments (Figure 4). 

### 3.3. Effect of Streptomyces *spp.* on Disease Development in Olive Plants

**Figure 5:**
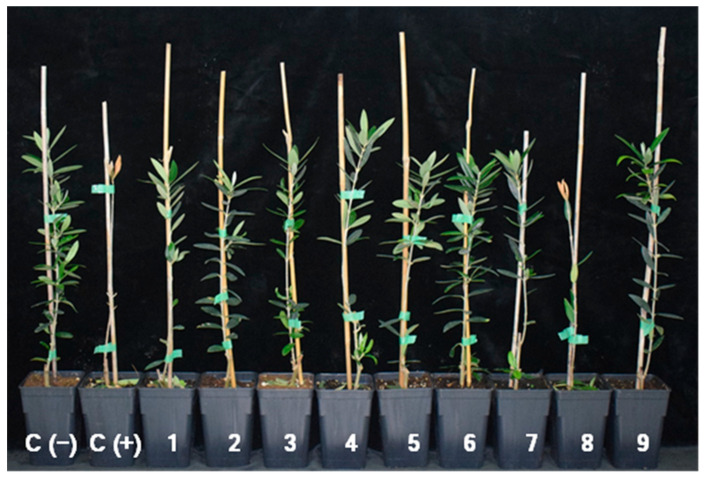
Disease symptoms on nine-month-old plants of cv. Picual at 3 months after inoculation with *Verticillium dahliae* isolate V323 and treated via irrigation with the biological control agents tested in this study. From left to right, untreated and non-inoculated control (**C (−)**), untreated and inoculated (**C (+)**), and treated plants with *Streptomyces* spp. strains CBQ-B-8 (**1**); CBQ-CB-14 (**2**); CBQ-CD-24 (**3**); CBQ-EA-2 (**4**); CBQ-EA-12 (**5**); CBQ-EBa-21 (**6**); CBQ-EA-2 + CBQ-B-8 (**7**); *Aureobasidium pullulans* AP08 (**8**); and *Fusarium oxysporum* FO12 (**9**).

There were significant differences between BCA treatments in DI (*p* ≤ 0.0001), mortality (*p* ≤ 0.0001), final DS (*p* = 0.0020), and RAUDPC (*p* = 0.0042). Considering all the disease-related parameters tested, AP08 showed significantly less effectiveness compared to the remaining treatments, with similar levels of DI, mortality, DS, and RAUDPC to those observed for the positive control; whereas the reference BCA FO12 was among the most effective treatments. At the end of the experiments, all the *Streptomyces* treatments showed DI values up to 50% compared to the negative control (DI = 0.0%), with DI ranging from 60.0 (CBQ-CB-14; CBQ-EBA-21) to 80.0% (CBQ-B-8; CBQ-EA-12). Only treatments with *Streptomyces* strains CBQ-CD-24 and CBQ-EA-2 and FO12 showed null mortality at the end of the experiment; whereas the mortality for the remaining *Streptomyces* treatments ranged from 6.7 (CBQ-CB-14; CBQ-EA-12; CBQ-EBA-21; CBQ-EA-2 + CBQ-B-8) to 13.3% (CBQ-B-8). Regarding final DS, most of the *Streptomyces* treatments and FO12 showed DS values below 1.0, with *Streptomyces* CBQ-EBa-21 (Final DS = 0.2 ± 0.03) and FO12 (Final DS = 0.3 ± 0.13) being the most effective. A similar pattern was observed for RAUDPC since CBQ-EBa-21 (DS = 4.0 ± 0.94%) and FO12 (DS = 15.7 ± 9.9) also showed the highest effectiveness in reducing disease progress compared to the remaining treatments and control (Table 3; Figure 5).

*Verticillium dahliae* was isolated successfully from the inoculated plants. The percentage of isolation ranged between 47.6 and 95.2% for lots of inoculated plants treated with *Streptomyces* sp. CBQ-CB-14 and AP08, respectively. The isolation percentage of *V. dahliae* from untreated and inoculated plants (positive control) was 61.9%, while the pathogen was not isolated from untreated and non-inoculated plants (negative control).

## 4. Discussion

The current demand for potential BCAs as an alternative for the control of phytopathogenic fungi is of great importance not only to reduce the agrochemical load deposited in ecosystems but also because it would contribute significantly to the preservation and conservation of natural ecosystems. Within this framework, the lack of effective chemical measures to prevent *V. dahliae* infections in olive trees, together with the restrictions on the use of chemicals by the European commission, makes it necessary to look for eco-friendly alternative for disease control. In this way, the present study reports the potential of *Streptomyces* strains as BCAs against VWO. 

It is worth mentioning that the six *Streptomyces* strains used in this study were selected because they were among the most effective in inhibiting the mycelial growth of the soilborne pathogens *M. phaseolina* and *R. solani* from a collection of 60 strains [42]. In addition, these strains, as well as the mix CBQ-EA-2 + CBQ-B-8, resulted in higher effectiveness in reducing the root rot diseases of *Phaseolus vulgaris* under both greenhouse and field conditions [42,58]. For this reason, the mix CBQ-EA-2 + CBQ-B-8 was also included in this study as an additional treatment. Due to these promising results, we hypothesized that these *Streptomyces* treatments deserved to be evaluated as potential BCAs against VWO.

The identity of the six *Streptomyces* strains (CBQ-B-8, CBQ-CB-14, CBQ-CD-24, CBQ-EA-2, CBQ-EA-12, and CBQ-EBa-21) was confirmed by sequencing the 16S and 23S rRNA genes and by phylogenetic analysis using single alignments. Our strains shared high similarity (97.00–99.78%) with reference sequences of *Streptomyces* available in the NCBI database, but none of the phylogenetic analyses were able to cluster our strains with specific *Streptomyces* species under consistent BS values. Consequently, the six strains were considered as unidentified species belonging to the *Streptomyces* genus, and they were classified as *Streptomyces* sp. Although we can find many studies in the literature in which the authors are able to identify *Streptomyces* species by only sequencing the 16S rRNA gene [59,60,61], our results agree with Khadayat et al. [62], since these authors indicate that identifying *Streptomyces* species is not possible by means of only 16S and 23S rRNA sequences due to the close relationship between species. They suggest analysis of acid methyl esters, metabolite profiles, and DNA–DNA hybridization to improve the identification of *Streptomyces* species [62]. Nevertheless, considering the difficulty in finding specific reference sequences that matches well with our strains, we consider that they are probably novel *Streptomyces* species. Thus, further research is needed in order to determine this aspect and, in such cases, to describe them as new species.

Regarding the effect of the biological treatments against *V. dahliae* viability, all the BCAs tested, i.e., the six *Streptomyces* strains, FO12, and AP08, showed a significant effect on the MGI of *V. dahliae* compared to the positive control. FO12 was the most effective on MGI, while the *Streptomyces* sp. strains showed an intermediate effect. Several studies have demonstrated the potential of *Streptomyces* spp. against a broad diversity of soil-borne fungal pathogens [30,31,42,58]. However, only a few studies have reported the effect of *Streptomyces* spp. against *V. dahliae*. In this way, our results agree with those obtained by Xue et al. [35], who showed that the strains *S. cyaneofuscatus* ZY-153, *S. kanamyceticu* B-49, *S. rochei* X-4, and *S. flavotricini* Z-13 had a significant effect on the MGI of *V. dahliae* (D–J), with the MGI ranging from 17.0 to 85.6% for ZY-153 and B-49, respectively, compared to the positive control. Similarly, Bubici et al. [34] showed that several *Streptomyces* spp. Strains (StB-3, StB-6, StB-11, and StB-12) inhibited the mycelial growth development of *V. dahliae*. Chen et al. [63] also tested the effect of *S. globisporus* strains Act7 and Act28 on *V. dahliae*, with the MGI ranging between 70.6 and 80.4%. Recently, Carlucci et al. [39] showed that *S. albidoflavus* strain CARA17 inhibited the mycelial growth of *V. dahliae*, with the MGI ranging from 87.42 to 71.30%. 

Regarding the effect of *Streptomyces* on the viability of *V. dahliae* MS, our results agree with those obtained by Xue et al. [64], who showed that culture filtrates of several *Streptomyces* sp. strains (B49, D184, and Act12) inhibited 100% of the germination of *V. dahliae* MS. In addition, other authors have shown that *Streptomyces* spp. strains were able to significantly reduce the viability of *V. dahliae* MS compared to the control [65,66]. The effect of our *Streptomyces* spp. strains in suppressing the viability of *V. dahliae* MS could be caused by the bioactive antifungal compounds produced by these strains, such as albocycline. Thus, our hypothesis is supported by the recent study conducted by Calvo-Peña et al. [36], who recently described the ability of albocycline to suppress the germination of *V. dahliae* conidia. However, more research in needed in order to determine the potential antifungal bioactive compounds produced by our *Streptomyces* spp. strains to elucidate their mode of action against *V. dahliae*.

The in planta bioassays revealed that irrigation treatments with *Streptomyces* were effective in significantly reducing the DS and disease progress in olive plants inoculated with *V. dahliae*, with the *Streptomyces* strain CBQ-EBa-21 being the most effective, together with the reference BCA FO12. However, the mix CBQ-EA-2 + CBQ-B-8 did not show any synergistic effect against the disease progress compared to the treatments with single *Streptomyces* strains. These results are not in concordance with those obtained by Diaz-Diaz et al. [42,58], who demonstrated that this mix had a significantly higher effect on reducing the DI and DS of root rot in *P. vulgaris* compared with single treatments with the *Streptomyces* sp. strains CBQ-EA-2 or CBQ-B-8. Unfortunately, there are not too many studies available with which to compare our results from in planta experiments with other studies focused on preventing *V. dahliae* infections by *Streptomyces* spp.; only Chen et al. [63] reveal that different inoculum sources of *Streptomyces* strains Act7 and Act28 applied by cotton seed treatments can significantly reduce Verticillium wilt in cotton seedlings. 

Finally, concerning the reference BCAs included in this study, the results on the effect of FO12 against *V. dahliae* were in concordance with those from previous studies conducted by Mulero-Aparicio et al. [20], and it was also the most effective treatment in our study, both in in vitro and in planta conditions. However, the results obtained for AP08 were in contrast with those shown by López-Moral et al. [5], since these authors revealed a low effect of AP08 against the pathogen in vitro, which coincides with our study, but a high effectiveness of AP08 in reducing the disease severity in inoculated olive plants. This contradictory effect could be associated with the difference in virulence of *V. dahliae* isolates, since different isolates were used in each study, or because it would require a greater number of treatments before inoculation with *V. dahliae* since AP08 acts by inducing resistance in the plant [40].

Thus, this study represents the first approach to elucidating the potential effect of *Streptomyces* sp. treatments against VWO. The high effectiveness of some strains, such as CBQ-EBa-21, in reducing the severity of VWO similarly to FO12, make it necessary to continue elucidating its mechanisms of action, i.e., antagonism, antibiosis, host-induced resistance, volatile organic compounds, etc. All these complementary experiments will be useful to better understanding how they act against the pathogen, and they will prove a relevant step in making these BCAs more attractive for private companies in the development of promising biological treatments against soil-borne pathogens.

## 5. Conclusions

The six *Streptomyces* strains used in this study were identified as *Streptomyces* sp. according to the 16S and 23S rRNA gene sequences, and they are considered potential new species that can be described in the future. All the BCAs tested showed a significant effect on mycelial growth and in the inhibition of germination of *V. dahliae* MS compared to the control, with the reference BCA *F. oxysporum* FO12 being consistently the most effective, while the *Streptomyces* sp. strains showed a moderate effect. Irrigation treatments with *Streptomyces* strain CBQ-EBa-21 were significantly more effective compared to the remaining treatments in reducing disease severity and disease progress in olive plants inoculated with *V. dahliae*. This study represents the first approach to elucidating the potential effect of *Streptomyces* sp. treatments against VWO, with promising results towards the development of new biological treatments.

## Figures and Tables

**Table 1:** *Streptomyces* strains used in the phylogenetic analysis and their corresponding GenBank accession numbers.

*Streptomyces* Species	Strain	Host	Origin	Collection Date	GenBank Acc. Number	
					16S rRNA	23S rRNA
*Streptomyces albidoflavus*	UST040711-291	- *	-	2009	FJ591130	-
	CCOS 2040	Grasslands soil	Switzerland	2009	-	OX371414
	SM254	Deep subsurface	USA	2010	-	CP014485
	RSU1	Mangrove soil	India	2012	KP698738	
	IHBA_9992	Suraj Tal Lake Water		2015	KR085950	-
	TN10	Termite nest	India	2017	MH021968	-
	IMB16-111	Marine sediment	China	2017	MG190667	-
	UYFA156	Seed *Festuca arundinacea*	Uruguay	2014	-	CP040466
	NBRC 13010	-	-	-	NR041095	-
	J1074	-	-	-	-	DS999645
*S. albogriseolu*	LBX-2	Soil	China	2012	-	CP042594
*S. aquilus*	GGCR-6	*Xanthium sibiricum*	China	2017	-	CP034463
*S. argenteolus*	CGMCC 4.1693	-	-	-	EU048540	-
*S. aurantiacus*	JCM 4677	-	-	-	-	AP023440
*S. aureoverticillatus*	HN6	Soil	China	2015	-	CP048641
*S. avermitilis*	MA-4680	-	-	-	-	NR076331
*S. calvus*	DSM 41452	-	-	-	-	CP022310
*S. caviscabies*	8	*Solanum tuberosum*	-	2016	KU743151	-
*S. champavatii*	173855	-	-	2008	EU570590	-
*S. coeruleorubidus*	ATCC 13740	Soil	-	-	-	CP023694
*S. cyanogenus*	S136	Soil/Goa mountain	India	1985	-	CP071839
*S. daghestanicus*	ICN1054	Marine	India	2010	KX775313	-
*S. deccanensis*	KCTC 19241	-	China	2019	-	CP092431
*S. dengpaensis*	XZHG99	Soil	China	-	-	CP026654
*S. exfoliatus*	3-11	Yalu River	North Korea	2014	KJ571038	-
	USC006	Fresh water	Australia	-	KX358629	-
*S. fodineus*	TW1S1	Soil	South Korea	2015	-	CP017248
*S. griseus*	NBRC 13350	Soil	-	-	-	NC010572
	MS098	-	-	2009	GU169072	-
*S. hydrogenans*	IMB16-184	Marine sediment	China	2017	MG190777	-
	IMB16-142	Marine sediment	China	2017	MG190741	-
*S. koyangensi*	VK-A60T	Soil	South Korea	2005	-	CP031742
	SCSIO 5802	Deep sea sediment	China	2016	-	CP049945
	B003	Sediment	-	2019	MG188671	-
*S. krainskii*	RSU51	Mangrove soil	India	2012	KP698745	-
*S. lividans*	RSU26	Mangrove soil	India	2012	KP698743	-
	XQ42	*Euphausia superba*	China	2012	KU291358	-
	T38	Soil	China	2016	KU317912	-
*S. nodosus*	ATCC 14899	Soil	-	-	-	CP023747
*S. phaeoluteigriseus*	Qhu-M197	Soil	China	2019	-	CP099468
*S. rutgersensis*	NBH77	Soil	Antarctica	2017	-	CP045705
*S. violascens*	KLBMP 5501	*Ginkgo biloba* L.	-	2015	KP636799	-
	IMB16-161	Marine sediment	China	2017	MG190757	-
	IMB16-135	Marine sediment	China	2017	MG190734	-
	ATCC 27968	Marine soil	China	2017	-	CP029377
	EGI125	-	-	2019	MN704434	-
*S. scabiei*	87.79	-	-	-	-	AB041112
*S. sampsonii*	KJ40	Soil	South Korea	2005		CP016824
	GACK10	Soil	-	2015	KP970678	-
*S. seoulensis*	HEK131	Soil	Japan	2014	-	AP025667
*S. somaliensis*	IMB16-131	Marine sediment	China	2017	MG190730	-
*Streptomyces* spp.	CBQ-B-8	Rhizosphere, Carbonated brown	Cuba	2008	OM417234	OR246887
	CBQ-CB-14	Sediment	Cuba	2013	OR251853	OR246888
	CBQ-CD-24	Rhizosphere	Cuba	2014	OR251854	OR246889
	CBQ-EA-2	Endophytic, stem of *Mosiera bullata*	Cuba	2008	OM417233	OR246890
	CBQ-EA-12	Endophytic, leaf of *Mosiera bullata*	Cuba	2011	OR251856	OR246891
	CBQ-EBa-21	Endophytic, root of *Piper aducum*	Cuba	2011	OR251857	OR246892
	IMB16-161	Marine sediment	China	2017	MG190757	-
	IMB16-135	Marine sediment	China	2017	MG190734	-
	ATCC 27968	Marine soil	China	2017	-	CP029377
*S. sudanensis*	MRC006	Mycetoma patient	Sudan	2016		JAJQLO000000000

* -: non-determined/non-available.

**Table 2:** Effect of biological control agents (BCAs) on the mycelial growth of *Verticillium dahliae* in dual cultures in vitro.

BCA Species	BCA Strain	MGI (%) ^a^
*Streptomyces* spp.	CBQ-B-8	42.8 ± 1.50 c
	CBQ-CB-14	42.3 ± 1.05 c
	CBQ-CD-24	37.3 ± 1.31 cd
	CBQ-EA-2	44.4 ± 1.67 c
	CBQ-EA-12	33.6 ± 2.91 d
	CBQ-EBa-21	39.0 ± 2.89 cd
*Aureobasidium pullulans*	AP08	52.1 ± 1.83 b
*Fusarium oxysporum*	FO12	76.4 ± 1.87 a

^a^ Mycelial growth inhibition (MGI, %) was obtained after growing both the BCAs and *V. dahliae* (isolate V004 or V323) in dual cultures on PDA at 28 °C for 14 days in the dark. Data represent the average of 12 replicated Petri dishes for each BCA or control ± the standard error of the means, corresponding to two experiments, two *V. dahliae* isolates, and three replications each. Means followed by a common letter do not differ significantly according to Fisher’s protected LSD test at *p* = 0.05 [55].

**Table 3:** Disease-related parameters for olive plants grown in artificially infested substrate with *Verticillium dahliae* isolate V323 and treated by irrigating with 250 mL of a spore suspension of FO12 or AP08 adjusted at 10^6^ conidia mL^−1^ and *Streptomyces* strains at 10^8^ spores mL^−1 a^.

Treatment (BCAs)	Isolate	Incidence (%) ^b,e^	Mortality (%) ^b,e^	Final DS ^c,f^	RAUDPC (%) ^d,f^
*Streptomyces* spp.	CBQ-B-8	80.0 bc	13.3 abc	1.2 ± 0.49 bc	55.1 ± 22.9 ab
	CBQ-CB-14	60.0 cd	6.7 c	0.6 ± 0.19 cd	18.8 ± 6.6 bc
	CBQ-CD-24	73.3 cd	0.0 d	1.1 ± 0.18 cd	34.7 ± 2.2 bc
	CBQ-EA-2	66.7 cd	0.0 d	0.9 ± 0.10 cd	22.9 ± 2.5 bc
	CBQ-EA-12	80.0 bc	6.7 c	0.9 ± 0.41 cd	27.5 ± 11.7 bc
	CBQ-EBa-21	60.0 cd	6.7 c	0.2 ± 0.03 d	4.0 ± 0.94 c
	CBQ-EA-2 + CBQ-B-8	73.3 cd	6.7 c	1.0 ± 0.12 cd	30.7 ± 1.0 bc
*Aureobasidium pullulans*	AP08	100 a	26.7 a	2.2 ± 0.39 a	92.8 ± 12.1 a
*Fusarium oxysporum*	FO12	66.7 cd	0.0 d	0.3 ± 0.13 d	15.7 ± 9.9 bc
Negative control	-	0.0 e	0.0 d	0.0 ± 0.0 e	0.0 ± 0.0 d
Positive control	-	93.3 ab	20.0 a	2.0 ± 0.41 ab	100 ± 39.5 a

^a^ Olive plants were treated before and after inoculation, and disease parameters were assessed weekly for 12 weeks after inoculation with *V. dahliae*. ^b^ Percentage of plants showing symptoms of the disease (Incidence) or death (Mortality) at 12 weeks after planting in the infested substrate with *V. dahliae* isolate V323. ^c^ Final disease severity (DS) ± standard error of the mean (SE) at 12 weeks after planting in the infested substrate with *V. dahliae* isolate V323. Disease severity was assessed using a rating scale of 0 to 4 (0 = no symptoms; 4 = 100% of affected plant tissues) expressed relative to the positive control. ^d^ Relative area under the disease progress curve (RAUDPC) ± SE developed over the assessment period. ^e^ In each column, data represent the means of 30 replicated plants. Means followed by a common letter do not differ significantly according to multiple comparisons tests for proportions at *p* = 0.05 [56]. ^f^ In each column, data represent the means of 30 replicated plants. Means followed by a common letter do not differ significantly according to Fisher’s protected LSD test at *p* = 0.05 [55].

## Data Availability

The data that support the findings of this study are available from the corresponding author upon reasonable request.
